# The Darker Facets of Systemic Lupus Erythematosus (SLE)

**DOI:** 10.7759/cureus.63176

**Published:** 2024-06-26

**Authors:** Gaffoor Codabaccus, Wiranthi Gunasekera

**Affiliations:** 1 Internal Medicine, Peterborough City Hospital, Peterborough, GBR; 2 Rheumatology, Peterborough City Hospital, Peterborough, GBR

**Keywords:** multi-disciplinary care, young adult ischemic stroke, sle, lupus nephritis, cerebral lupus

## Abstract

Cerebrovascular events remain a rare but serious feature of systemic lupus erythematosus (SLE). In this report, we see a 25-year-old lady who presented with sudden-onset right-sided weakness and speech disturbances. She was initiated on anti-platelet therapy and glucocorticoids. Her admission was complicated by worsening kidney function due to lupus nephritis. She responded well to immunosuppressant therapy and was discharged following resolution of her symptoms for outpatient specialist follow-up. The rarity of such cases poses a diagnostic and treatment challenge. A language barrier and difficult social circumstances can exacerbate this. However, awareness of neuropsychiatric lupus as a differential diagnosis at the acute assessment of stroke and early involvement of specialist teams, allied health professionals, and safeguarding teams can lead to a successful long-term outcome.

## Introduction

Systemic lupus erythematosus (SLE) is a chronic autoimmune inflammatory disease. This is associated with serological markers such as antinuclear antibody (ANA), double-stranded DNA antibody (dsDNA), and a variety of other extractable nuclear antigens. It can affect almost any organ, with common symptoms including rash, oral ulcers, photosensitivity, alopecia, arthritis, and fatigue [1]. Neuropsychiatric (NP) involvement in SLE can be independent of the presence of other serological or systemic disease manifestations. The symptoms of NP lupus can vary from anxiety or mild cognitive impairment to more serious and life-threatening features such as stroke and seizures [2].

The prevalence of NP symptoms remains highly variable due to its broad definition, with figures ranging from 14.0% to 80.0% in adults, out of which 16.5-33.9% were directly attributed to SLE [3]. Cerebrovascular events remain one of the most severe NP presentations of SLE with a prevalence of 3-20% and account for up to 15% of SLE-related deaths [2]. Furthermore, a hospital-based study showed that the yearly incidence of undiagnosed SLE patients presenting with a stroke is 2.4 per 100.000 people (aged 20-24), 4.5 (aged 30-34), and 32.9 (aged 45-49) [4]. A meta-analysis of cohort studies done by Holmqvist et al. showed that risks of a cerebrovascular event such as stroke or intracranial hemorrhage were higher in patients with SLE, especially in those under the age of 50 [5]. Secondary causes of ischaemic stroke need to be investigated in these patients, which will include excluding conditions such as patent foramen ovale, carotid artery dissection, arrhythmias, and antiphospholipid syndrome [5,6].

When it comes to the multi-organ involvement of SLE, a 3-year prospective study done by Kampylafka et al. in 2013 showed that only 4.3% of the 370 patients in the study had major central nervous system involvement, out of which 44% had concurrent renal involvement and 63% had hematological manifestations with the NP lupus [7]. Given the rarity and severity of this condition, early identification and initiation of treatment remains one of the key features in dealing with such patients. Once the diagnosis is made, treatment modalities will involve a combination of antiplatelet, glucocorticoid, and immunosuppressant therapy [3].

## Case presentation

A 25-year-old lady presented to the emergency department with a 24-hour history of pleuritic chest pain, right-sided weakness, paraesthesia, facial droop, dysarthria, and dysphasia. She was an immigrant who spoke limited English with no fixed abode due to some social concerns and consequently was not registered with a local general practitioner. Her past medical history was significant for SLE, which was diagnosed at the age of 11 in her country of origin. She was meant to be on maintenance treatment of hydroxychloroquine 200 mg once daily and prednisolone 10 mg daily, but, unfortunately, she was poorly compliant with her treatment.

Her physical examination revealed a National Institute of Health Stroke Scale (NIHSS) score of 8 due to a right-sided reduction in power and sensation, alongside a right facial droop, dysphasia, and dysarthria. She also had a soft pansystolic murmur, which was audible over the mitral area. A computed tomography (CT) scan of the head was promptly done, which showed no acute hemorrhage, so she was started on 300 mg of aspirin for a probable acute ischaemic stroke. She was reviewed by the rheumatology team given the history of poorly controlled SLE, and she was thoroughly investigated for potential causes of stroke in a young adult (Tables 1-2).

**Table 1:** Laboratory investigations. 1. Quantitative values of ANA, Anti-dsDNA, C3, and C4 levels were indicators of the severity of the disease. Improvements in the numbers and the clinical picture were indicative that the patient was responding appropriately to the treatment. 2. Viral screen included testing for HIV, hepatitis, Epstein-Barr virus (EBV), and cytomegalovirus (CMV). 3. Lumbar puncture was done to test for probable bacterial and/or viral infections. 4. Detailed analysis of the renal biopsy results: Congo red stain negative for amyloid. The capillary basement membrane is irregularly thickened with small to medium granular electron-dense deposits present in all aspects. Both epithelial and endothelial cells are enlarged, focal podocyte foot process effacement is seen, and occasional tubuloreticular inclusions are present in endothelial cells. The tissue submitted for immunofluorescence did not contain any glomeruli. Immunoperoxidase has been performed. This shows granular glomerular basement membrane expression with IgG, IgM, C1q, and C3. IgA shows weak positive staining in the same pattern of distribution. 5. International Society of Nephrology/Renal Pathology Society.

Marker	Results on admission	Results prior to discharge	Normal range
Haemoglobin	71	124	115–165 (g/L)
White cell count	2.9	8.9	4.0–11.0 (10^9^/L)
Platelets	115	285	150–400 (10^9^/L)
Creatinine	68	131	45–84 (umol/L)
ANA^1^	>200	>200	<20 (CU)
Anti-dsDNA^1^	>379 (Crithidia positive)	97	0–10 (IU/mL)
C4^1^	<0.03	0.26	0.14–0.54 (g/L)
C3^1^	0.35	0.83	0.75–1.65 (g/L)
Ferritin	2071	1206	30–400 (ug/L)
Antiphospholipid antibodies	Negative	-	-
Anti-Ro antibody	Positive	-	-
Haemophilia screen	Negative	-	-
Viral screen^2^	Negative	-	-
Lumbar puncture^3^	Negative	-	-
Urine dipstick	-	Protein ++++ Blood ++++ Glucose, Nitrites, Ketones negative	-
24-hour urine analysis	-	2.6	<0.1 (g/24 hours)
Urine protein-creatinine ratio	-	369	<45 (g/mol)
Renal biopsy^4^	-	ISN/RPS^5^ Class IV lupus nephritis	-

**Table 2:** Imaging results. 1. MRI Head implies magnetic resonance imaging of the head without contrast. 2. MR angiogram intracranial implies magnetic resonance angiography of the intracranial arteries.

Imaging modality	Results
CT Head on Admission	No acute intracranial haemorrhage
MRI Head^1^	Predominantly gyriform acute infarction in the left frontoparietal region and insular cortex (Figure 1). Multiple small foci of haemosiderin signal suggestive of sequela of previous microhaemorrhages (Figure 2). Features are suggestive of CNS manifestations of SLE, possible vasculitis
MR Angiogram Intracranial^2^	Tiny saccular aneurysm noted over the right anterior cerebral artery. No carotid or vertebral artery dissections noted
Echocardiogram	Mild mitral regurgitation. No features of Liebmann-Sacks endocarditis
CT Pulmonary Angiogram	Negative for pulmonary embolism

**Figure 1:**
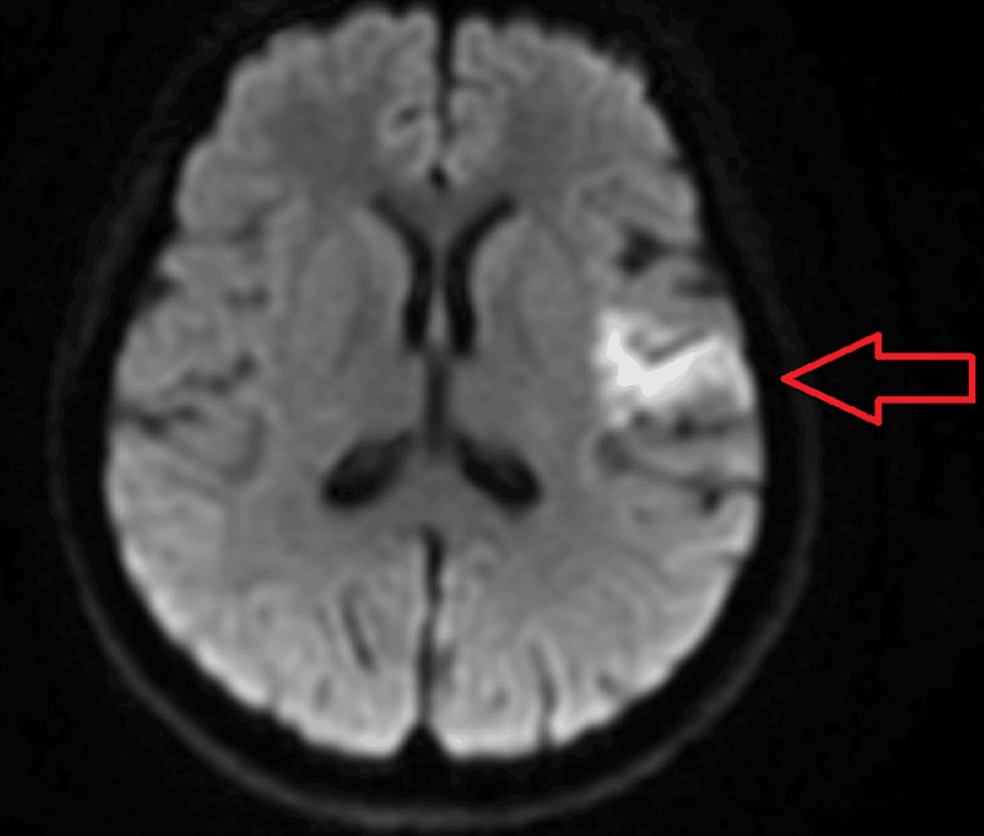
Diffusion-weighted imaging (DWI) sequence of the MRI showing acute infarction in the left frontoparietal region and insular cortex.

**Figure 2:**
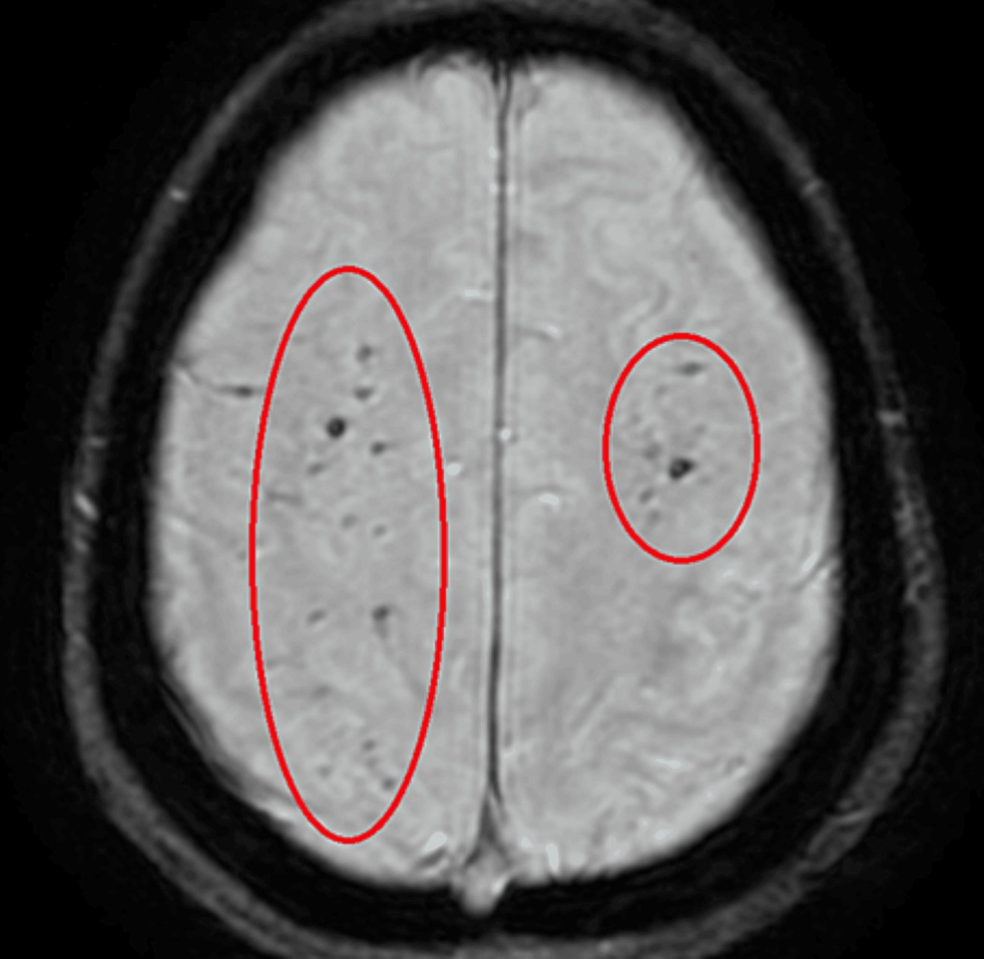
Susceptibility-weighted angiography (SWAN) sequence of the MRI showing the sequela of previous microhaemorrhages.

With features of cerebral lupus identified on her MRI Head (Figures 1-2) alongside her significantly deranged auto-immune panel (Table 1), a diagnosis of NP lupus was promptly established. Her SLEDAI 2K score was calculated at 24, thereby highlighting the severity of her current presentation. She was consequently started on high doses of intravenous methylprednisolone, followed by a tapered course of oral prednisolone.

During the daily ward round reviews, it was noted that she developed new persistent hypertension with bilateral worsening pedal oedema and dropping albumin levels. This was accompanied by a worsening kidney function test. A urine dipstick revealed significant microscopic haematuria and proteinuria, so a 24-hour urine analysis and a urine protein-creatinine ratio were done, which revealed nephrotic range proteinuria. She was consequently referred to the nephrology team for consideration of a renal biopsy. In order to ensure the safest approach for the procedure, she was discussed with the stroke physician and the nephrologists who collectively agreed on a plan to hold the aspirin for seven days before and after the procedure, which allowed for an uneventful biopsy with no complications. The biopsy confirmed the diagnosis of lupus nephritis, which was deemed as Class IV as per the ISN/RPS classification.

In order to induce remission, she was given two infusions of rituximab, 1 g each, and given two weeks apart, followed by mycophenolate mofetil 1 g orally twice daily for maintenance. She remained on low-dose aspirin for secondary stroke prevention. With physiotherapy and speech therapy input, her dysarthria and dysphasia resolved within a week. The right-sided weakness, facial droop, and kidney function tests resolved completely within the following eight weeks.

The language barrier and her difficult social circumstances were the main challenges to her treatment. With the input of interpreters, safeguarding and social services teams we were able to support her care. The multidisciplinary team enabled us to communicate clearly, establish trust, discuss health beliefs, and gain informed consent. This practice also established a safe environment for long-term outpatient treatment.

## Discussion

We presented the case of a young lady with SLE who suffered from multi-organ complications as a result of her poor compliance with her initial maintenance treatment. Literature has shown that almost every organ can be affected in this condition, especially in patients with poor compliance, who may even present with life-threatening conditions such as stroke or seizures [1,2]. However, this case also showed how prompt recognition and initiation of treatment can potentially lead to the reversibility of the complications listed earlier.

Upon initial presentation, our patient was managed as an acute stroke with high-dose aspirin therapy. Thrombolysis was not considered given her late presentation to the Accident and Emergency Department. As illustrated by previous articles on managing patients with central nervous system (CNS) involvement of lupus [2,6], we also investigated for secondary causes of ischaemic stroke in a young adult, such as arrhythmias, structural heart disease, and antiphospholipid syndrome (APL), among others. Once APL was ruled out, we continued treating our patient as per the management flowchart outlined in the 2019 article by Nikolopoulos et al. [2], which recommended treatment with a single anti-platelet alongside high dose methylprednisolone, followed by a tapering dose of oral prednisolone. The latter guidance also recommended adding immunosuppressants to the above therapy when multi-organ involvement is suspected, which is why our patient was pulsed with rituximab and initiated on mycophenolate mofetil once lupus nephritis was suspected.

The above treatment combinations triggered a slow but significant symptomatic improvement in her clinical picture. Her limb weaknesses started to improve as did her dysphasia and facial droop. Alongside monitoring for clinical improvement, the guidance also recommends monitoring the quantitative values of the patient’s ANA, anti-dsDNA, complement levels, and renal function tests as a guide to assess the efficacy of the treatment [3]. Table 2 shows how the biochemical markers also showed improvement in our patient. Moreover, a multidisciplinary team involving the stroke physician, rheumatologist, nephrologist, physiotherapist, and occupational therapist was set up to ensure the best possible care for the patient as recommended in the article by Piga et al. in 2011 [6]. This enabled us to ensure adequate follow-up, which resulted in a near-total recovery of her neurological deficits and clinical picture.

## Conclusions

NP lupus can be a common manifestation of patients in SLE, especially in younger patients. The symptoms of NP lupus can vary from anxiety to mild cognitive impairment, stroke, and seizures. When a stroke is suspected in a patient with SLE, it is important to exclude secondary causes such as carotid artery dissection, patient foramen ovale, antiphospholipid syndrome, or arrhythmias. In such patients, early identification and initiation of treatment have been associated with good short-term prognosis. Patients diagnosed with SLE, especially those diagnosed at a younger age, should therefore be educated about the signs and symptoms of a cerebrovascular event and encouraged to seek help if they develop any concerning symptoms. In acute strokes, the use of MRI remains a more sensitive tool than CT for identifying the changes in cerebral involvement of SLE. Once a diagnosis of NP lupus is established, the management would include symptomatic and immunosuppressant therapy alongside a multi-disciplinary team approach.
